# Data on the sensory characteristics and chemical composition of the edible red seaweed dulse (*Palmaria palmata*) after dry and semi-dry storage

**DOI:** 10.1016/j.dib.2020.106343

**Published:** 2020-09-29

**Authors:** Pierrick Stévant, Aðalheiður Ólafsdóttir, Paul Déléris, Justine Dumay, Joël Fleurence, Bergrós Ingadóttir, Rósa Jónsdóttir, Émilie Ragueneau, Céline Rebours, Turid Rustad

**Affiliations:** a Møreforsking AS, PO Box 5075, 6021 Ålesund, Norway; b Norwegian University of Science and Technology NTNU, 7491 Trondheim, Norway; c Matís ohf, Vínlandsleið 12, 113 Reykjavík, Iceland; d MMS (Mer Molécule Santé), EA2160, Université de Nantes, BP 92208, 44322 Nantes, France

**Keywords:** Seaweed, Sensory analysis, Volatile compounds, Storage, Processing, Nutritional composition, Physico-chemical properties, Flavor compounds

## Abstract

The data article refers to the paper “Semi-dry storage as a maturation process for improving the sensory characteristics of the edible red seaweed dulse (*Palmaria palmata*)” [1]. The data refers to the analysis of samples of the edible seaweed species *Palmaria palmata* during storage in a dry (D, containing ca. 6 % moisture) and semi-dry state (SD, containing ca. 20 % moisture). The article includes data from the analysis of samples taken at 0, 12, 61 and 126 days of storage to evaluate the effect of moisture content and storage time on the sensory characteristics of the product. The variations in flavor, odor and texture between samples were measured by sensory evaluation. Data from the analysis of flavor-active compounds (free amino acids and volatile compounds), macronutrient content (soluble proteins and carbohydrates, lipid and mineral fractions), physico-chemical properties (water activity, water and oil-binding capacities, swelling capacity), color and microbial load are also reported. The information provided in this article can be used by industrial stakeholders (seaweed producers, food industry) to optimize processing and storage conditions of edible seaweeds and by scientists to build upon further knowledge to improve the quality of seaweeds in food applications.

## Specifications Table

**Table:** 

Subject	Agricultural and Biological Sciences
Specific subject area	Food science
Type of data	Table Image Figure
How data were acquired	Gravimetric method for the determination of moisture and ash contents. Sensory analysis: generic descriptive analysis [2] of the samples following 12 attributes, using a panel of 9 judges trained according to standard guidelines [3]. Water activity: measured with a LabMaster-aw (Novasina AG, Lachen, Switzerland). Water-soluble proteins and carbohydrates: solid-liquid extraction using sodium phosphate buffer (1/20, w/v), protein content quantified using the BCA reagent assay (Thermo Fisher Scientific, Waltham, MA, USA). R-Phycoerythrin determined spectrophotometrically from measured absorbance at 455, 565 and 592 nm and using the Beer and Eshel equation [4]. Carbohydrates quantified spectrophotometrically using the phenol-sulfuric method [5] with modifications. Total nitrogen: quantified by the Kjeldahl method [6]. Free amino acids: solid-liquid extraction in distilled water followed by HPLC analysis (Water Novapak C18 column and RF 2000 fluorescence detector, Dionex, Sunnyvale, CA, USA). Protein electrophoresis: SDS-PAGE method (Protean 3 system, BIO-RAD, Hercules, CA, USA) followed by colloidal Coomasie staining of the gels. Lipids: extraction in dichloromethane and methanol (2/1, v/v) followed by filtration, phase separation and gravimetric quantification. Physico-chemical properties: water and oil incorporation followed by centrifugation. Color: capture of digital images of the samples, color data collection using an image processing program (Adobe Photoshop). Microbial load: plating sample homogenates followed by enumeration of total viable counts. Volatile compounds: extraction on SPME fiber (Supelco, Bellefonte, PA, USA), analysis by GC-MS on a ZB-5MS column (Phenomenex, Torrance, CA, USA) and measurement using Shimadzu Q2010 GS-MS system.
Data format	Raw data Analyzed data
Parameters for data collection	Biomass of *P. palmata* was wild harvested and dried. Semi-dry test samples were partially rehydrated to ca. 20 % moisture while dry samples did not receive any treatments. All samples were sealed then stored in the dark at 12°C. Samples were taken at 12, 61 and 126 days of storage, freeze-dried and stored at -80°C until analysis. All treatments (moisture level, storage duration) were tested in triplicate.
Description of data collection	The variations in flavor, odor and texture between samples were measured by sensory evaluation. The water activity and moisture content of the samples and their macronutrient content was analyzed, including soluble proteins and carbohydrates, lipid and mineral fractions. Their composition in flavor-active compounds (free amino acids and volatile compounds) were determined as well as their physico-chemical and color characteristic. Some of the samples (at the end of the storage period) were analyzed for their microbial status.
Data source location	Møreforsking AS, PO Box 5075, 6021 Ålesund, Norway
Data accessibility	With the article
Related research article	Stévant P, Ólafsdóttir A, Déléris P, Dumay J, Fleurence J, Ingadóttir B, Jónsdóttir R, Ragueneau É, Rebours C, Rustad T (2020) Semi-dry storage as a maturation process for improving the sensory characteristics of the edible red seaweed dulse (*Palmaria palmata*). Algal Research 51:102048. https://doi.org/10.1016/j.algal.2020.102048

## Value of the Data

• The data is useful to describe the changes in key food-quality parameters of *P. palmata* during dry and semi-dry storage i.e. sensory properties, chemical composition, flavor-active compounds, physico-chemical characteristics and microbiological status.
• The data provided in this article can be used by seaweed producers and the food industry to optimize processing and storage conditions of seaweeds and develop seaweed-based products.
• The data can be used by scientists to build upon further knowledge to improve the quality of seaweeds in food applications.
• This is the first available data reporting changes in flavor and chemical content of an edible seaweed species during storage, in relation to the moisture content of the material.

## 1 Data Description

This Data in Brief article provides figures and data sets from the sensory analysis of dry (D) and semi-dry (SD) samples of *P. palmata*, their water activity and chemical composition including free amino acid (FAA) and volatile compounds composition, physico-chemical, color characteristics and microbiological status.

Table 1 reports the results from the generic descriptive analysis of the samples, averaged scores over panelists (*n* = 9) on a scale from 1 to 100, based on 12 attributes describing flavor (F), odor (O) and texture (T). The mean scores of the samples (*n* = 3) for each attribute is reported in the spider plot in Fig. 2. The attributes are listed and described in the Table 1 of the related research article [1]. The data from the analysis of water activity (a_w_), moisture content (MC), soluble proteins and carbohydrates, R-phycoerythrin (R-PE), lipids, ashes and total nitrogen (total N) of the samples is summarized by the boxplots in Fig. 3. The gels from protein electrophoresis of the samples are displayed in Fig. 4. Fig. 5 reports the mean values, from the analysis of the FAA composition of the samples. Fig. 6 describes the data from the determination of the water- and oil-binding capacity (WBC and OBC) and swelling capacity (SC). Data from the color analysis of the samples is reported in Fig. 7. Table 2 reports the raw data from the microbial analysis of the D and SD samples at the end of the experiment. Table 3 displays processed data for peak area from the analysis of volatile compounds of duplicate seaweed samples by gas chromatography-mass spectrometry (GC-MS). Raw data tables from the chemical characterization of the samples i.e. MC, a_w_, soluble carbohydrates and proteins, R-PE, lipid, ashes, total N and FAA composition, as well as raw data from the determination of WBC, OBC, SC and color profiles are available as supplementary material in Microsoft Excel Worksheet format. These data are comprehensively discussed in the reference article [1] except for the color characteristics and protein electrophoresis which were not included in the original article.

**Table 1:** Averaged scores over panelists (*n* = 9) on a scale from 1 to 100, from the generic descriptive analysis of *P. palmata* samples after dry (D) and semi-dry (SD) storage over a period of 126 days, based on 12 attributes describing flavor (F), odor (O) and texture (T).

Sample	Replicate	O-seaweed	O-sweet	O-hay	O-fish skin	F-salty	F-seaweed
D-126	1	39.44	25.56	29.67	37.89	52.89	47.22
	2	34.56	32.11	32,44	30.11	61.89	43.44
	3	47.89	33.00	30,89	19.22	67.33	36.56

SD-12	1	41.78	31.33	43,33	17.11	58.00	42.00
	2	30.22	31.00	31,33	29.56	57.78	36.33
	3	42.44	26.89	33,00	21.78	63.44	38.67

SD-61	1	29.67	42.11	41,44	11.67	61.00	32.11
	2	32.00	40.89	52,89	10.44	56.22	33.22
	3	34.11	33.67	47,11	12.11	57.67	36.22

SD-126	1	28.67	39.33	45,78	12.33	57.56	31.67
	2	34.11	39.89	54,11	8.56	69.44	33.22
	3	30.56	41.44	43,44	10.67	62.44	39.67


Sample	Replicate	F-richness	F-processing	F-dried fish	F-bitter	T-crunchy	T-tough
D-126	1	35.00	27.89	23.89	8.56	59.44	59.00
	2	40.44	35.00	22.22	9.89	55.78	67.67
	3	43.11	34.22	21.11	7.11	56.78	56.11

SD-12	1	44.44	36.67	20.67	7.22	45.67	50.56
	2	42.89	33.67	18.00	12.67	56.78	60.89
	3	44.00	36.89	16.56	7.00	52.33	65.44

SD-61	1	52.33	43.11	12.44	7.67	42.44	43.78
	2	44.78	44.78	13.44	10.11	45.00	52.78
	3	44.00	41.11	11.00	5.11	46.00	46.78

SD-126	1	43.00	38.67	16.33	12.33	42.22	36.89
	2	47.56	47.00	12.56	8.78	43.11	39.89
	3	40.89	38.44	14.89	11.44	47.89	40.89

**Table 2:** Microbial load (expressed in CFU g^−1^) measured at the surface of dry (D) and semi-dry (SD) samples of *P. palmata* stored for 126 days.

Sample	Aerobes	Molds	Yeasts	Coliforms
D-126	16000	450	<10	<10
D-126	8000	830	<10	<10
D-126	7200	350	<10	<10

SD-126	260	<10	<10	<10
SD-126	1400	20	<10	<10
SD-126	16000	<10	<10	<10

**Table 3:** Peak area (10^−6^) from the analysis of volatile compounds from *P. palmata* samples after dry (D) and semi-dry (SD) storage over a period of 126 days.

Compound name	Retention time	D-126	D-126	SD-12	SD-12	SD-61	SD-61	SD-126	SD-126
*Alcohols* 1-Penten-3-ol	3.87	4.3	4.5	4.9	4.7	6.6	4.6	4.9	4.9
1-Octen-3-ol	15.77	6.3	5.3	10.4	6.1	12.6	9.2	11.6	11.4
1-(2-methoxy-1-methylethoxy)propan-2-ol	16.56	4.8	4.7	7.1	8.0	3.8	2.7	4.2	3.7
1-(2-Methoxypropoxy)-2-propanol	16.93	7.4	7.8	15.4	10.2	4.1	n.d.	4.1	6.7
Benzyl alcohol	17.58	3.5	4.2	3.0	2.7	3.0	n.d.	4.7	3.3
2,4,4-trimethylcyclohex-2-en-1-ol	18.12	2.2	3.2	7.5	3.0	7.8	7.2	8.1	12.6
2,6-Dimethylcyclohexanol	19.57	6.7	7.7	30.4	9.4	33.0	22.9	36.4	41.2
3-Cyclohexene-1-ethanol	21.29	2.7	3.1	6.6	3.2	6.6	9.6	6.3	7.7
2-amino-4-methoxyphenol	21.92	n.d.	n.d.	4.3	n.d.	4.2	6.0	7.6	8.8
2,3,6-trimethyl-7-Octen-3-ol	22.62	2.3	2.7	4.0	n.d.	3.9	4.3	n.d.	n.d.
1-(1-Adamantyl)-1-phenylethanol	23.92	n.d.	n.d.	2.5	n.d.	2.4	3.6	2.9	3.5

*Aldehydes* 3-Methylbutanal	3.40	1.6	2.3	2.0	1.5	2.5	1.7	2.0	2.1
Hexanal	7.32	4.3	3.4	7.1	4.9	8.3	9.8	7.8	8.7
(E)-2-Hexenal	9.56	2.0	4.5	8.2	5.1	8.9	8.9	7.4	8.6
(Z)-4-Heptenal	11.78	2.2	2.4	6.4	2.9	7.3	7.0	7.7	7.4
Heptanal	11.90	4.9	3.8	3.1	3.6	3.6	3.8	3.6	4.4
Benzaldehyde	14.83	7.2	12.3	9.7	9.2	12.3	7.8	8.9	11.3
Octanal	16.62	6.3	4.8	5.4	4.7	5.8	7.4	7.3	9.6
(E,E)-2,4-Heptadienal	16.87	4.2	5.0	14.4	7.1	12.3	18.6	11.9	11.1
(E)-4-Oxohex-2-enal	17.51	n.d.	n.d.	3.4		3.6	4.6	6.3	7.3
Benzeneacetaldehyde	17.85	2.6	2.4	2.4	1.9	2.4	2.8	2.9	3.3
Nonanal	19.44	19.5	12.7	19.2	16.1	19.6	22.1	20.1	23.3
(E,Z)-2,6-Nonadienal	20.46	4.3	5.1	10.3	5.8	9.7	13.8	10.4	10.7
Decanal	21.47	6.9	6.5	9.6	7.2	9.7	11.4	11.0	15.7
2,6,6-trimethyl-cyclohexene-1-carboxaldehyde	21.75	3.1	3.7	5.8	3.4	6.8	4.7	5.2	6.0
Undecanal	23.14	5.0	4.0	3.1	4.6	2.8	3.8	4.1	4.6

(E,E)-2,4-Decadienal	23.33	n.d.	n.d.	2.7	1.4	2.6	4.5	3.0	3.8
3,5,5-Trimethyl-2-hexene	15.51	25.6	29.5	38.4	29.8	48.2	23.4	39.1	36.7
7-Methyl-3-octyne	17.45	n.d.	n.d.	2.2	n.d.	2.6	2.1	2.5	2.8
3-Dodecyne	22.52	6.3	8.1	22.0	8.9	20.7	22.8	21.4	24.3
1-butenylidene-cyclohexane	22.76	2.3	2.9	3.3	2.7	3.2	4.0	3.0	3.9
Cyclohexene, 3-(3-methyl-1-butenyl)-, (E)-	24.31	1.5	1.9	n.d.	2.4	2.9	2.8	n.d.	n.d.
Tetradecane	24.47	3.4	4.1	2.5	3.8	2.4	2.0	n.d.	3.0
Pentadecane	28.22	22.7	17.1	7.6	8.2	6.6	7.1	7.1	8.8
Heptadecane	25.81	88.1	90.7	110.0	96.9	104.9	73.0	102.7	90.7
Diethylacetic acid	21.69	n.d.	n.d.	4.1	4.3	3.5	4.0	2.0	2.5
Bis[2-(trimethylsilyl)ethyl] malonic acid	22.95	11.7	11.1	11.0	11.0	11.3	11.9	11.7	12.1

3,5,5-Trimethylcyclohex-3-en-1-one	18.69	2.9	3.5	n.d.	n.d.	n.d.	n.d.	n.d.	n.d.
3,5-Octadien-2-one	19.17	6.2	6.7	21.0	7.0	24.7	19.9	20.6	21.8
3,4,4-trimethyl-2-cyclopenten-1-one	19.40	n.d.	n.d.	3.3	n.d.	3.5	3.2	5.4	5.8
2,6,6-Trimethyl-2-cyclohexene-1,4-dione	20.31	n.d.	n.d.	4.1	1.7	4.6	4.7	6.8	8.0
alpha-Ionone	24.87	6.8	9.5	22.2	9.6	21.8	22.4	25.6	37.9

n.d.: non detected.

## 2 Experimental Design, Materials, and Methods

### 2.1 Experimental design

**Fig. 1:**
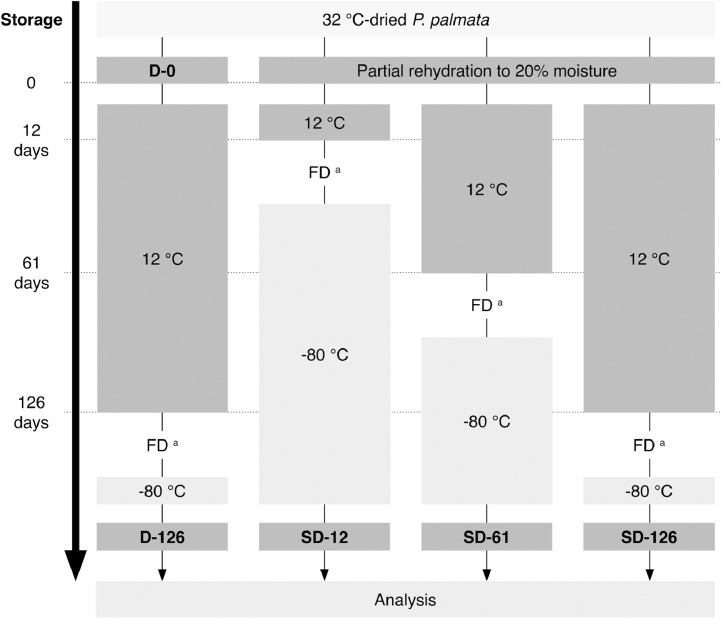
Experimental scheme to study the effects of dry (D) and semi-dry (SD) storage on the sensory properties, chemical composition, physico-chemical characteristics and microbial status of *P. palmata*. The samples were stored in sealed bags, in the dark at 12°C. Freeze-drying (FD) then vacuum-packing and storage at -80°C were used to stabilize and prevent further reactions within the samples. All samples consisted of 3 replicates. ^a^ Samples were analyzed for their MC, a_w_, color and microbiological status in their original form (i.e. dried or semi-dried), prior to FD.

Wild biomass of *P. palmata*, free from epiphytes, was harvested at Roscoff in France in November 2017 (Biocean, France) and air-dried at 32°C in a shelf-dryer for 24 h. At reception to the laboratory, the MC of the material was measured, and the biomass divided into 2 batches: a semi-dry (SD) and a dry (D) control group, each comprising 3 sample replicates of 900 g and 650 g respectively. The SD-samples were partially rehydrated by spraying the required amount of water (unfiltered tap water) on the seaweeds to achieve a MC of 20 %, as preliminary tests showed microbial stability of *P. palmata* at this level of moisture. The D-samples did not receive any treatment at reception to the laboratory. All samples were sealed in polyethylene bags (not vacuumed) then stored in the dark at a constant temperature of 12°C. Samples of the SD-group were taken after 12, 61 and 126 days of storage (labelled SD-12, SD-61 and SD-126 respectively) and were freeze-dried, vacuum-packed and stored at -80°C until analysis. D-samples were taken at reception to the laboratory (D-0) and after 126 days of storage (D-126) under the conditions described above. The chemical and physico-chemical analyses were conducted on freeze-dried samples, to exclude the potential bias of comparing samples of different MC. Only the MC, water activity (a_w_) and microbial analyses were performed on the samples in their original form. Fig. 1 provides a schematic overview of the experimental design.

### 2.2 Sensory analysis

**Fig. 2:**
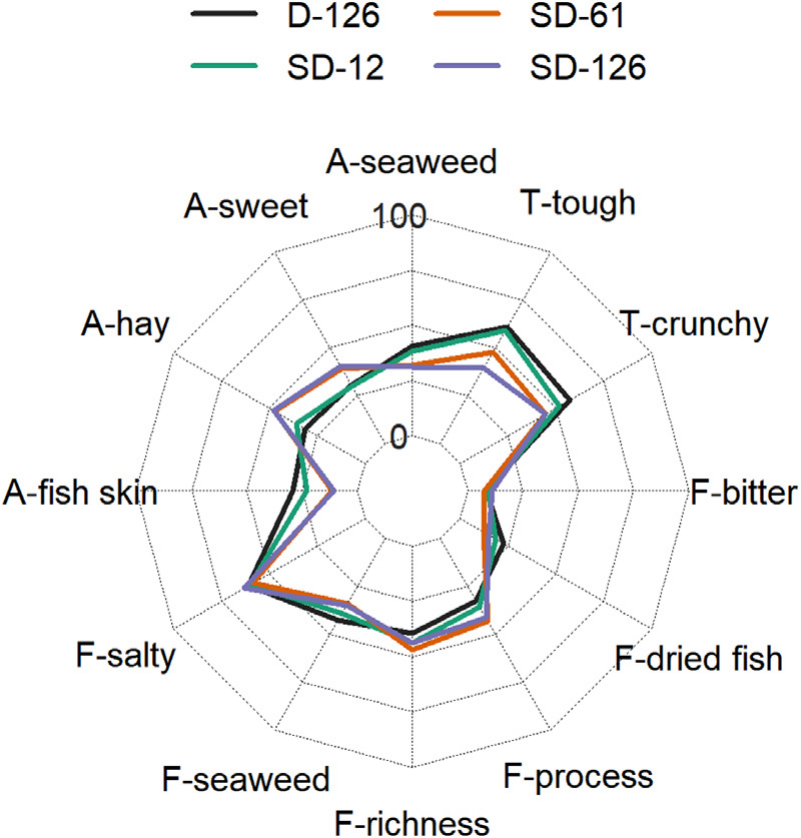
Mean scores for aroma (A), flavor (F) and texture (T) characteristics from the generic descriptive analysis of *P. palmata* samples after dry (D) and semi-dry (SD) storage over a period of 126 days.

A generic descriptive analysis [2] was used to characterize and compare the sensory profiles of SD- (SD-12, SD-61, SD-126) and D-126 samples of *P. palmata*. The sensory panel consisted of 9 judges selected from the staff at Matís ohf and trained according to the guidelines in ISO:8586 [3]. All assessors had some experience with sensory evaluation of seaweeds. All samples, i.e. including the dry control group, were rehydrated to 20 % MC prior to the evaluation to avoid the potential bias of evaluating samples of different MC [7]. Three panel training sessions were carried out prior to the evaluation. During the first two training sessions, a scale was developed for the *P. palmata* samples, based on scales from earlier experiments with seaweeds [7], [8]. In the third training session, sensory attributes were further defined, and the use of the scale was synchronized between the panelists. In each training session, two to three samples were used as references. The final vocabulary consisted of twelve attributes to describe the odor, flavor and texture characteristics of the samples. During the sensory evaluation, the intensity of each attribute for a given sample was described using a 15-cm unstructured scale which was transformed to numbers from 0 to 100 (lowest to highest intensity) for the data analysis. Both the training and sensory evaluation phases were conducted in a sensory test facility equipped with individual booths. Red lights were used during the evaluation to mask any possible differences in the appearance of the samples. Four samples, coded with three-digit numbers, were evaluated in each of the three replicate sessions. The samples were presented to the panel as individual portions (ca. 3 g) in a white plastic cup with a lid. The sensory evaluation program FIZZ (2.50B, Biosystèmes, France) was used to collect sensory data. The program Panelcheck (V1.4.0, Nofima, Norway) was used to evaluate the performance of the sensory panel and individual panelists. The Table 1 reports the average scores over panelists (*n* = 9) for each sample for the selected attributes. The Fig. 2 displays the mean scores (*n* = 3) for each treatment.

### 2.3 Moisture, a_w_ and chemical characterization

***Moisture and a_w_*** The MC of both D- and SD-samples was determined gravimetrically by drying ca. 5 g of sample at 105°C for 24 h (until constant weight). 3 measurements were conducted on each sample replicate. The subsequent results from chemical analyses were expressed as part of the DW of the samples. The a_w_ was measured with a LabMaster-aw (Novasina AG, Lachen, Switzerland).

***Ash*** Ash content was determined after combustion of the dried samples at 590°C for 12 h in a laboratory muffle furnace (Type 62700, Barnstead Thermolyne, Ramsey, MN, USA). The ashes were quantified gravimetrically as the residue from combustion. 3 measurements were conducted on each sample replicate.

***Water-soluble carbohydrates and proteins*** Crude extracts were obtained from ground samples in liquid nitrogen homogenized with sodium phosphate buffer (20 mM, pH = 7.1) at a 1/20 ratio (w/v) under stirring for 20 min at 4°C. After centrifugation at 25000 *g* at 4°C for 20 min, the resulting supernatant contained the water-soluble compounds. Three replicate extracts were obtained from each sample. The content of water-soluble carbohydrates were analyzed using the modified colorimetric phenol-sulfuric acid method [5]. Phenol at 5 % (200 μL) was added to 200 μL of extract or glucose solution followed by 1 mL of sulfuric acid (96 %). The solutions were allowed to stand for 10 min at room temperature before vortexing (10 sec at 2000 *g*), then 15 min at room temperature and 30 min at 35°C (in a water bath) before the absorbance was measured at 490 nm. Glucose was used as a standard. The protein content of the extracts was quantified using the bicinchoninic acid (BCA) protein reagent assay (Thermo Fisher Scientific, Waltham, MA, USA) according to the protocol of Smith et al. [9]. Bovine serum albumin was used as protein standard. The R-phycoerythrin (R-PE) content was determined spectrophotometrically using the Beer and Eshel [4] Eq. (1) and the measured absorbance (*A*) from the extracts at 455, 565 and 592 nm:

(1) $$\left[R-PE\right]=\left[\left(A_{565}-A_{592}\right)-\left(A_{455}-A_{592}\right)\times 0.20\right]\times 0.12$$

***Total nitrogen*** The nitrogen content was quantified by the Kjeldahl method [6] and an estimate of the total protein content was calculated by multiplying the nitrogen content by a factor of 5 as considered suitable to predict the protein content of seaweeds.

***Lipids*** The lipid content of the samples was determined according to the method of Bligh and Dyer [10] with modifications. Freeze-dried samples ground in liquid nitrogen were rehydrated at a 1/4 ratio (w/v) with ultrapure water. Lipids were extracted with a mixture of dichloromethane and methanol (2/1, v/v). The extract was filtered on fritted glass then KCl (0.88 %) was added to the filtrate to improve phase separation. The lipid content was determined gravimetrically. The analysis was conducted in three parallels on each sample replicate.

**Fig. 3:**
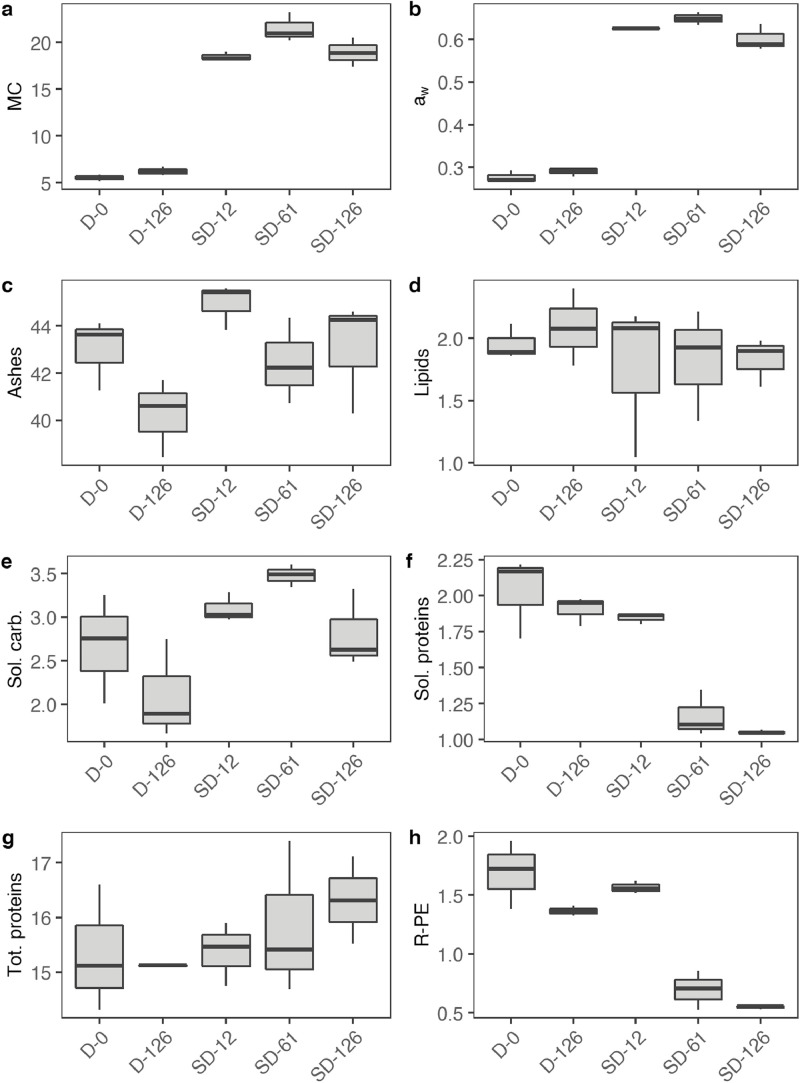
Boxplots of the a) moisture content (MC, in % of dry weight (DW)), b) water activity (a_w_, dimensionless) and chemical content i.e. c) ashes, d) lipids, e) soluble carbohydrates, f) soluble proteins, g) total proteins (N * 5) and h) R-phycoerythrin (R-PE) expressed in g (100 g)^−1^ DW of dry (D) and semi-dry (SD) samples of *P. palmata* during storage. For each sample, *n* = 9 except from a_w_ and total protein measurements (*n *= 3). Raw data tables are available as supplementary material.

The results from moisture, a_w_ and chemical characterization are presented in Fig. 3.

**Fig. 4:**
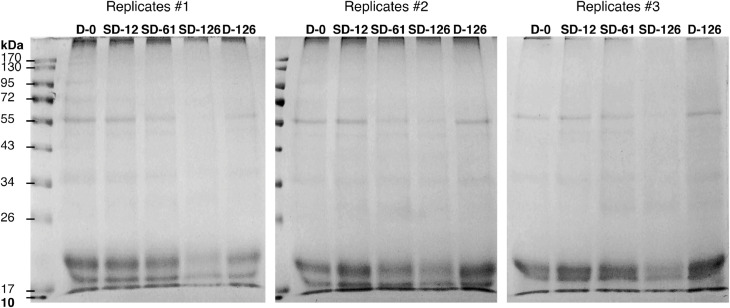
SDS-PAGE analysis of the soluble protein fractions of triplicate dry (D) and semi-dry (SD) samples of *P. palmata* during storage.

***Protein electrophoresis*** The samples were ground in liquid nitrogen and the resulting powder was homogenized with tris buffer 50 mM (pH 7.4) supplemented with the following protease inhibitors: EDTA 5 mM, phenylmethylsulfonyl fluoride (PMSF) 1 mM, pepstatin A 1 μM, bestatin HCl 10 μM and leupeptin hemisulfate 100 μM. Hydrosoluble protein were extracted by adding Triton X-100 1.5 % for a 1-h incubation under gentle shaking at 4°C. Proteins were concentrated by centrifugation (15 000 *g* at 4°C) on 3 kDa Vivaspin 500 concentrators (Sartorius, Goettingen, Germany). Protein content of the extracts were determined using the BCA assay kit as described above. The absorbance was measured in 96 well plates at 570 nm using an ELx800 UV universal microplate reader (Bio-Tek Instruments, Inc., Winooski, VT, USA). 40 μg of each sample was mixed with denaturing Laemmli buffer (containing β-mercaptoethanol) and loaded on sodium dodecyl sulfate-polyacrylamide (12 %) gel electrophoresis (SDS-PAGE). After migration on a Protean 3 system (BIO RAD, Hercules, CA, USA) the gels were submitted to highly sensitive colloidal Coomassie staining according to the protocol of Dyballa and Metzger [11]. Gels were scanned with a Geldoc XR (Thermo Fisher Scientific, Waltham, MA, USA). The gels obtained from the SDS-PAGE analysis of triplicate samples are represented in Fig. 4.

**Fig. 5:**
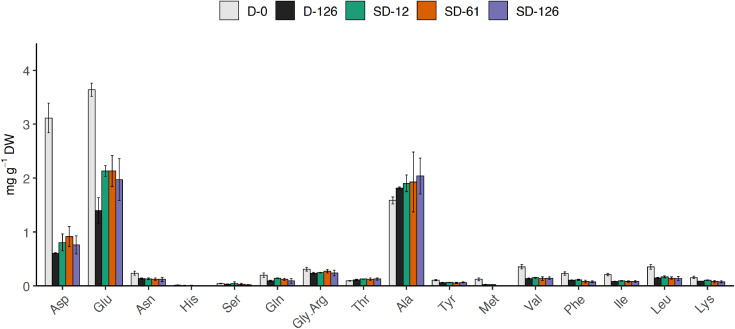
Free amino acid profile of dry (D) and semi-dry (SD) samples of *P. palmata* during storage. Values are given as mean ± standard deviation (*n *= 3). The raw data table is available as supplementary material.

***Free amino acids*** The FAA composition of the samples reported in Fig. 5 was determined using the method of Osnes and Mohr [12]. The proteins were extracted by agitating 0.8 g of ground dried sample in 10 mL distilled water for 1 h. The extract was centrifuged at 4°C and 12 000 *g* for 20 min. 0.25 mL of 10 % sulphosalicylic acid was added to 1 mL of the water-soluble extract. The mixture was then vigorously shaken and incubated at 4°C for 30 min prior to centrifugation at 7840 *g* for 10 min to precipitate the protein-bound amino acids. 1 mL of the supernatant was added to 0.25 mL of 10 % sulphosalicylic acid and the same operation as previously described was repeated until no protein precipitate was observed. Each sample was extracted in triplicate. Suitably diluted samples were filtered (0.2 μm) prior to duplicate analysis of each extract by high-performance liquid chromatography (HPLC) (Dionex Ultimate 3000) using a Water Novapak C18 column (4.0 μm particle size) and a RF 2000 fluorescence detector (Dionex, Sunnyvale, CA, USA). The FAAs were identified and quantified by comparison with pure amino acid standards purchased from Fluka (Buchs, Switzerland). Both cysteine and proline were excluded from the analysis, cysteine being unstable during the acid hydrolysis of the samples and proline undetected following the o-phtalaldehyde (OPA) pre-column derivatization during the HPLC analysis. The results were expressed in mg g^−1^ DW of the seaweed samples.

### 2.4 Physico-chemical parameters and color characteristics

***Water and oil binding capacity (WBC and OBC)*** WBC and OBC was determined by adding 30 mL of either distilled water or a commercial soya oil to 0.5 g ground samples (particle size 0.8 mm) in a 50-mL centrifuge tube. The samples were then stirred and left at room temperature for 1 h. After centrifugation at 3000 *g* for 20 min, the supernatant was discarded, and the residue weighed. WBC and OBC were expressed as gram water and oil per gram of dried sample. The WBC and OBC analysis of each sample was performed in three parallels.

**Fig. 6:**
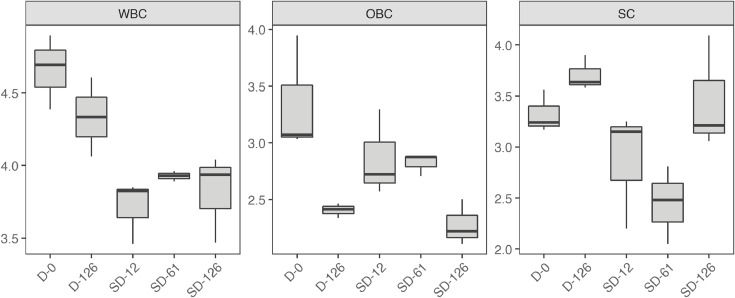
Water binding capacity (WBC, in g water g^−1^ (dry sample), oil binding capacity (OBC, in g oil g^−1^ (dry sample) and swelling capacity (SC, in mL g^−1^ (dry sample) of dry (D) and semi-dry (SD) samples of *P. palmata*. For each sample, *n* = 9 except from SC measurements (*n *= 3). The raw data table is available as supplementary material.

***Swelling capacity (SC)*** SC was determined by adding 1 to 2 g ground samples to a 50-mL measuring cylinder. 30 mL of distilled water was added under agitation using a vortex mixer to eliminated trapped air bubbles. The samples were covered and left overnight then SC was determined as the volume occupied by the sample (in mL) per gram of dry sample initially added. The results from the analysis of physico-chemical parameters of the samples are presented in Fig. 6.

**Fig. 7:**
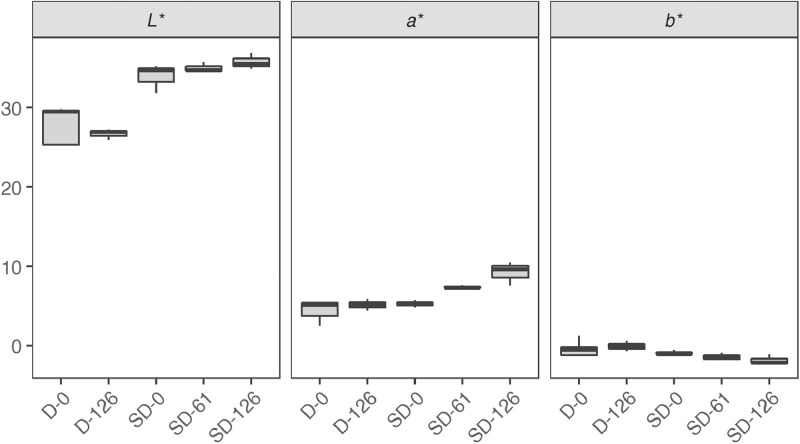
Color profile of dry (D) and semi-dry (SD) samples of *P. palmata* during storage considering the dimensionless coordinates L* (lightness), a* (redness/greenness) and b* (yellowness/blueness). For each sample, *n *= 15. The raw data table is available as supplementary material.

***Color*** The surface color profile of D-0, D-126, SD-61, SD-126 and SD samples after partial rehydration (labelled SD-0) was analyzed by a computerized image technique using a digital camera (Canon EOS 6D) and a 50-mm lens (Canon EF 50 mm f/1.4) mounted in a black box isolated from external light. The camera color profile was calibrated with a ColorChecker (X-Rite, Grand Rapids, MI, USA). Uniform and constant lighting was achieved using LED strips positioned at an angle of 45° from the sample to obtain uniform lighting. The color was analyzed quantitatively using Photoshop (Photoshop CC 2017, Adobe Systems Inc.) and expressed in CIE L* (whiteness or brightness), a* (redness/greenness), and b* (yellowness/blueness) coordinates, as described by Yam and Papadakis [13]. Color measurements of each sample replicate was performed in five parallels. The results from color analysis are presented in Fig. 7.

### 2.5 Microbial load

The microbial load of both D- and SD-samples at the end of the storage period (D-126 and SD-126) were analyzed. Approximately 5 g of each sample were diluted in a ratio 1:10 using peptone water (pH 7.0 ± 0.2) and homogenized in a stomacher (Seward Ltd, Worthing, UK). Five serial dilutions were then plated (1 mL) onto different types of count plates, namely aerobic, coliform, and yeast and mold count plates (3M Petrifilm, Maplewood, MN, USA). The incubation time was 72 h at 30 ˚C for aerobes, 24 h at 37 ˚C for coliforms and 48 h at 25 ˚C for yeasts and molds as validated by standard methods [14]. The total viable count (TVC) was enumerated following the manufacturer guidelines for each type of plate. The microbial load of the samples was expressed in colony forming unit (CFU) per g sample. Microbial analyses of the samples were conducted in triplicate. The microbial load of each samples replicate is reported in Table 2.

### 2.6 Analysis of volatile compounds by headspace solid phase microextraction (HS-SPME) and gas chromatography-mass spectrometry (GC-MS)

The extraction of volatile compounds of D-126 and SD-samples of *P. palmata* was carried out using a SPME fiber (65 μm polydimethylsiloxane–divinylbenzene, 23 Ga needles, StableFlex™) supplied by Supelco (Bellefonte, PA, USA). The fiber was conditioned before use at 270°C for 1 h and placed into the SPME adapter for a CTC autosampler (CTC Analytics, Zwingen Switzerland) fitted with a vial heater, according to manufacturer instructions. Two of three sample replicates in each group were analyzed and accurately weighed (1 g) into eight 20 mL headspace vials and the samples pre-incubated in vial heater for 15 min at 50°C. The samples were extracted for 30 min before injecting the fiber and desorbing in the GC injection port for 5 min at 230°C under splitless conditions as described by López-Pérez et al. [15]. The volatile compounds were separated on a ZB-5MS column, 30 m long, 0.25 mm internal diameter, 0.25 μm film thickness (Phenomenex, Torrance, CA, USA). Measurements were performed on a Shimadzu Q2010 GC-MS. Helium was used as a carrier gas and the temperature program was as follows; 35°C for 3 min, 35°C to 70°C at 3°C min^−1^, 70°C to 200°C at 10°C min^−1^, 200°C to 260°C at 20°C min^−1^ and held for 3 min. Injection temperature was 230°C and ion source was kept at 250°C. Interface temperature was 265°C. The mass detector was set to scan from 35 – 400 m/z. Tentative and qualitative identification of volatile compounds was performed by comparing mass spectra of peaks to the NIST's library (National Institute of Standards and Technology, Gaithersburg, MD, USA) based on the calculated degree of similarity (similarity index). All samples were evaluated using the same integration parameters, i.e. using peak height as set minimum. Among all volatile compounds detected in each sample, only those detected in both sample replicates were selected. It should be noted that the HS-SPME-GC-MS method used is not validated by analysis of known standards to fully confirm the identity and quantity of the detected volatiles. Limit of detection (LOD) and limit of quantification (LOQ) are calculated based on standard deviation (StD) of multiple measurements of blank samples, where LOD is 3 x StD and LOQ is 10 x StD. At LOQ the relative standard error (RSD) is 30%. Since the LOQ for this method is unknown, RSD between two replicate measurements was calculated for each detected compound.

## CRediT authorship contribution statement

**Pierrick Stévant:** Conceptualization, Methodology, Formal analysis, Investigation, Writing - original draft, Writing - review & editing, Visualization. **Aðalheiður Ólafsdóttir:** Methodology, Investigation, Writing - review & editing. **Paul Déléris:** Investigation, Writing - review & editing. **Justine Dumay:** Investigation, Writing - review & editing. **Joël Fleurence:** Methodology, Resources, Supervision, Writing - review & editing. **Bergrós Ingadóttir:** Investigation. **Rósa Jónsdóttir:** Resources, Writing - review & editing. **Émilie Ragueneau:** Investigation. **Céline Rebours:** Methodology, Resources, Supervision, Writing - review & editing, Project administration, Funding acquisition. **Turid Rustad:** Methodology, Resources, Supervision, Writing - review & editing.

## Declaration of Competing Interest

The authors declare that they have no known competing financial interests or personal relationships which have, or could be perceived to have, influenced the work reported in this article.

## Appendix Supplementary materials

Image, application 1
